# Multi-Input Regulation and Logic with T7 Promoters in Cells and Cell-Free Systems

**DOI:** 10.1371/journal.pone.0078442

**Published:** 2013-10-23

**Authors:** Sukanya Iyer, David K. Karig, S. Elizabeth Norred, Michael L. Simpson, Mitchel J. Doktycz

**Affiliations:** 1 Graduate Program in Genome Science and Technology, University of Tennessee, Knoxville, Knoxville, Tennessee, United States of America; 2 Biosciences Division, Oak Ridge National Laboratory, Oak Ridge, Tennessee, United States of America; 3 Center for Nanophase Materials Sciences, Oak Ridge National Laboratory, Oak Ridge, Tennessee, United States of America; 4 Department of Materials Science and Engineering, University of Tennessee, Knoxville, Knoxville, Tennessee, United States of America; 5 Center for Environmental Biotechnology, University of Tennessee, Knoxville, Knoxville, Tennessee, United States of America; Imperial College London, United Kingdom

## Abstract

Engineered gene circuits offer an opportunity to harness biological systems for biotechnological and biomedical applications. However, reliance on native host promoters for the construction of circuit elements, such as logic gates, can make the implementation of predictable, independently functioning circuits difficult. In contrast, T7 promoters offer a simple orthogonal expression system for use in a variety of cellular backgrounds and even in cell-free systems. Here we develop a T7 promoter system that can be regulated by two different transcriptional repressors for the construction of a logic gate that functions in cells and in cell-free systems. We first present LacI repressible T7lacO promoters that are regulated from a distal lac operator site for repression. We next explore the positioning of a tet operator site within the T7lacO framework to create T7 promoters that respond to tet and lac repressors and realize an IMPLIES gate. Finally, we demonstrate that these dual input sensitive promoters function in an *E. coli* cell-free protein expression system. Our results expand the utility of T7 promoters in cell based as well as cell-free synthetic biology applications.

## Introduction

Engineering synthetic gene circuits entails the redesign of existing gene networks or the creation of novel genetic functions to perform a predetermined task. Construction of these circuits has been valuable in attaining a bottom up understanding of biological systems[1] and offers potential for harnessing biological function for biotechnology[2,3] and biomedicine[4,5]. Well-characterized genetic components have been integrated into circuits that function as logic gates [6,7], memory elements, clocks[8] and counters. Ultimately, like their electronic analogues, components can be assembled into larger networks for practical applications in medicine, bioremediation[9] and chemical synthesis [10]. However, despite the fact that a rapidly growing number of gene circuits are being published, the complexity of the systems is not keeping pace[11]. One factor in this apparent complexity barrier is that gene circuits typically operate within cellular systems and rely on the host’s endogenous promoters and translational machinery to drive circuit function. Therefore, unintended interactions with endogenous processes can make implementation of predictable and rationally designed circuits difficult. Consequently, orthogonal expression systems that insulate the synthetic gene circuits from other biological networks are required[12-14]. 

One such orthogonal expression system exploits the mono-subunit T7 RNA polymerase, which is commonly utilized because of its simplicity, stability, processivity, and specificity. In contrast to multi-subunit bacterial RNA polymerases, T7 polymerase recognizes a specific 17 base pair promoter sequence and does not require co-factors to activate transcription [15]. T7 RNA polymerase is also highly processive and has been extensively used for achieving high yield protein expression[16,17]. Furthermore, since T7 RNA polymerase is highly specific for T7 promoters, its use permits exclusive expression from user-defined genes in a variety of different backgrounds without interference from endogenous promoters[18]. For these reasons, T7 promoters have been used in protein expression applications in both live cells and in cell-free systems. Therefore, T7 circuit elements will be valuable not just in cell based systems for achieving orthogonal expression but can also be used in cell-free systems to assemble circuits of increasing complexity [19-22]. 

However, a significant challenge to implementing complex gene regulatory circuits with T7 promoters is that, unlike bacterial promoters, the commonly used viral promoters have few natural mechanisms for activating or repressing gene expression. Synthetic T7 promoter variants can be repressed by transcription factors such as LacI and TetR only when bound to a relatively short regulatory region positioned proximal to and downstream from the transcription start site [16,23-27]. As a consequence of the short cis-regulatory region, engineering T7 promoters that respond to multiple transcriptional regulators is very challenging and few strategies regulation of the T7 promoter by multiple transcription factors have been described [28]. 

**Figure 1:**
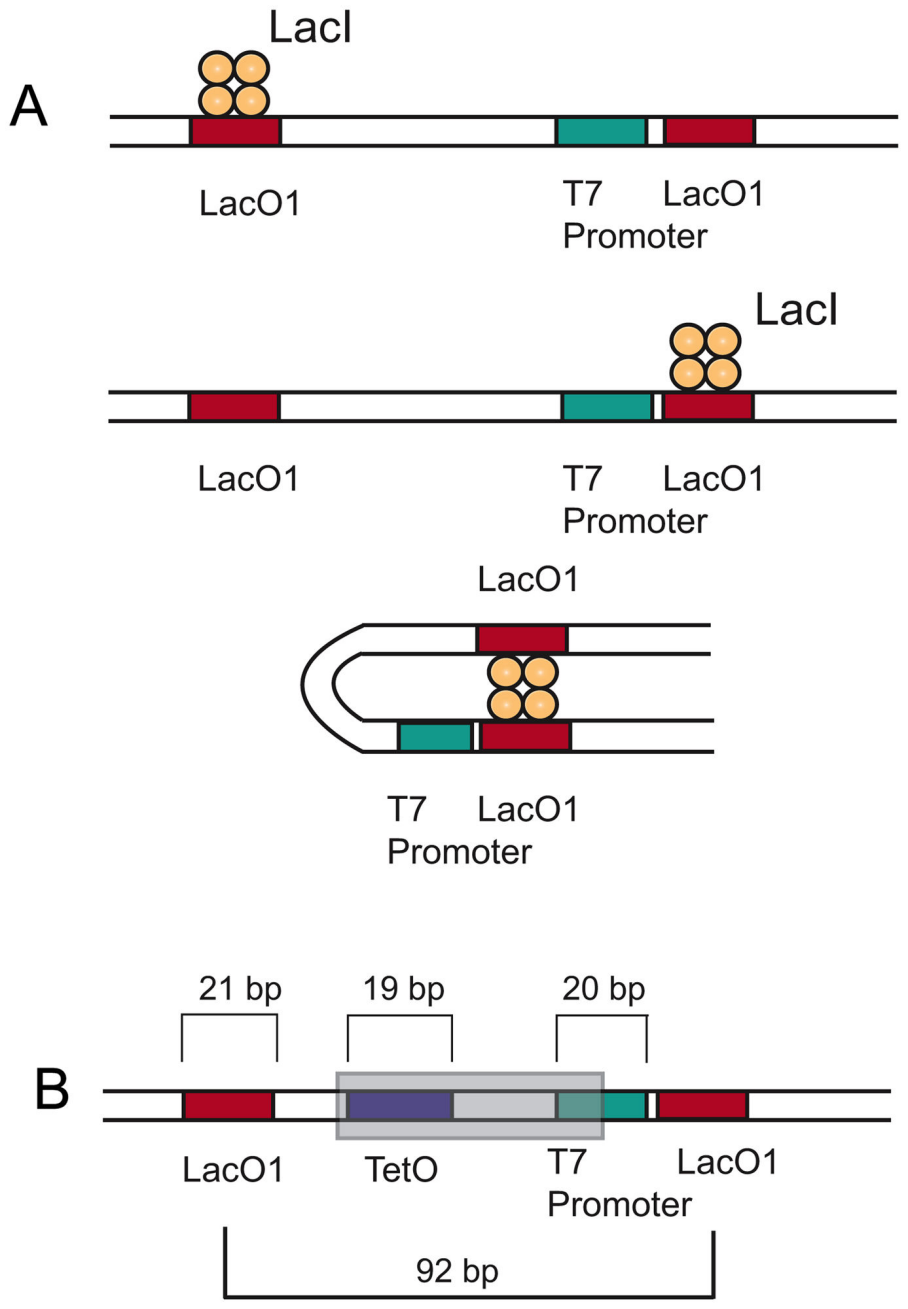
Design strategy for achieving combinatorial regulation of expression from T7 promoters. A) An auxiliary lacO is placed upstream to a conventional T7lacO promoter to create stronger LacI repressible T7 promoters. DNA looping is induced by the binding of a single LacI tetramer to both of the lacO binding sites. B) TetR binding regions (tetO) placed within this DNA looping framework, at regions indicated by grey box, can enable multi-input regulation by interfering with LacI mediated looping.

In this article, we develop an approach for engineering T7 promoters that can be regulated by multiple inputs, and we use this approach to implement a digital logic function. To achieve this, we first focused on extending the effective regulatory region of the T7 promoter. As an alternative to cis-regulation of T7 promoter, we harness DNA looping to enable regulation from distal locations. In natural systems, DNA looping can be mediated by protein multi-merization and is commonly used to enable transcription regulation by the synergistic action of repressors bound at different locations. For instance, Lac repressor proteins (LacI) bind their operators as a tetramer or a dimer of dimers. Native *E. coli* lac promoters contain auxiliary Lac operators (LacO) upstream and downstream to the *E. coli* lac promoter. At low LacI concentrations, presence of additional Lac operators induces DNA loop formation in the intervening DNA, thereby increasing the probability of LacI occupancy of the *E. coli* lac promoter [29,30] and enhancing repression from *E. coli* promoters. We show that, as with *E. coli* lac promoters, an improvement in repression of T7lacO promoters can be attained by appropriately spacing a second lac operator 92 bases upstream to a T7lacO promoter. We then demonstrate that this extension of the T7 regulatory region through DNA looping enables the development of multi-input regulation. Specifically, the binding of a second regulator, TetR, at a distal site interferes with the loop enhanced repression of the first regulator, LacI. We examine the effect of placement of tetO, the binding site for the TetR protein, into the LacI looping framework so as to generate T7 promoters that respond to both TetR and LacI (Figure 1). We found that Tet operators placed in between the two lac operators interfere with Lac mediated repression. 

Using our dual-input T7 promoters, we present an extendable strategy for creating T7-based logic gates. We demonstrate an IMPLIES logic gate which accepts TetR and LacI proteins as inputs. IMPLIES gates together with NOT gates can be used to perform any kind of logic operation, thereby providing a way for implementing logic functions of choice from T7 promoters[31]. Furthermore, we demonstrate the functionality of these TetR and LacI repressible T7 promoters in both live *E. coli* cells and in cell-free expression systems. 

## Materials and Methods

### Plasmids and Bacterial strains

All plasmids used in this study were constructed using standard molecular biology techniques and are listed in Table 1. DNA used in cell-free experiments was prepared using Qiagen Plasmid Midi prep kits or Biorad midi prep kits. Plasmids will be made available upon request. *E. coli* strain BL21-AI (Invitrogen Inc, WI) was used for protein purification and for live cell expression experiments. LB media with 100 μg/mL ampicillin was used to culture cells for protein purification and for preparation of starter cultures for live cell experiments. Minimal media for testing plasmids had the following composition: M9 salts with Casamino acids (Amresco), 2 mM MgSO_4_, 0.5% glycerol, 300 μM thiamine, and 100 μg/mL ampicillin.

**Table 1:** List of plasmids used in this study.

**Plasmid Name**	Promoter*	Operator, position**	**Gene**	**Backbone**
pREPT7 01	T7lacO	LacO1, +14	*EGFP*	pET3a
pREPT7 11	T7lacO	LacO1, +14, -77	*EGFP*	pET3a
pREPT7 31	T7lacO	LacO1, +14; LacO3, -77	*EGFP*	pET3a
pREPT7 ID1	T7lacO	LacO1, +14; LacOID, -77.5	*EGFP*	pET3a
pDRT7 77	T7lacO	LacO1, +14, -77; tetO, -44	*EGFP*	pET3a
pDRT7 14	T7lacO	LacO1, +14; tetO, -44	*EGFP*	pET3a
pTetRLacI	*E. coli* promoter	-	*tetR, lacI*	pPROLAR

pET3a has a copy number of approximately 40 [64], and pPROLAR has a copy number of 20-30 [65].

* The subscripts indicate the number of bases deleted from the T7 promoter from the - 17^th^ base.

** The operator position refers to the distance between the transcriptional start site (+ 1 base and the center of the operator sequence that binds the transcriptional repressor.

### Purification of TetR

TetR was purified as previously described. Briefly, BL21-AI *E. coli* cells (Invitrogen Inc, WI) harboring pET-TetRHis [24] were grown in LB media with 100 μg/mL ampicillin at 37°C and were induced using 0.2% L-arabinose. The cells were resuspended in binding buffer (50 mM sodium phosphate buffer, pH 8.0, 300 mM NaCl, 10 mM imidazole) and lysed by sonication. The supernatant obtained after centrifugation of the samples was applied to a Ni-NTA column. The column was subsequently washed with buffer (50mM sodium phosphate, pH 8.0, 300 mM NaCl, 50 mM imidazole). TetR-His6 was then eluted with elution buffer (50mM sodium phosphate, pH 8.0, 300 mM NaCl, 500 mM imidazole). Finally, the purified protein was concentrated and dialysed into the final storage buffer (20 mM sodium phosphate pH 7.2, 50 mM NaCl).

### GFP measurements from *E. coli* experiments

**Figure 2:**
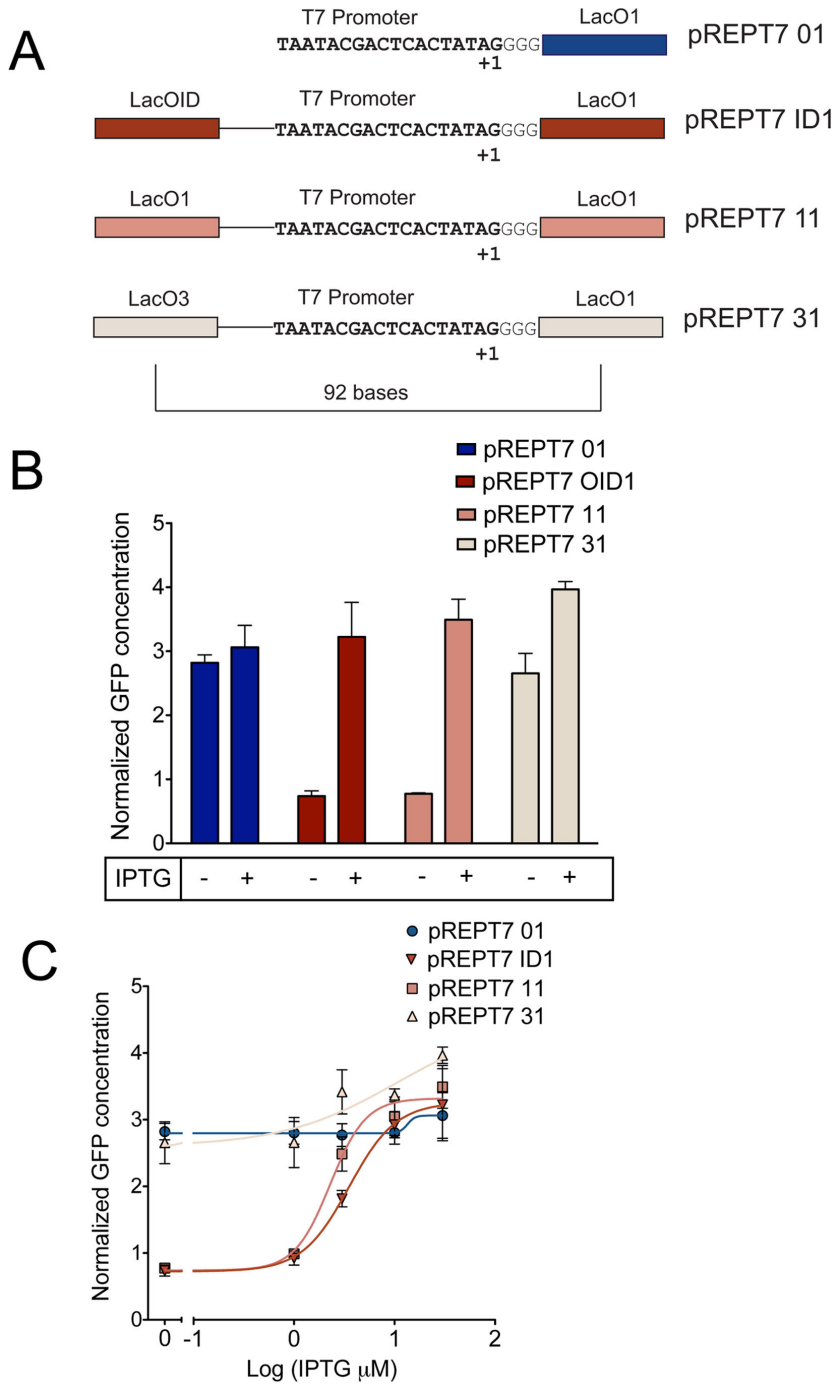
Effect of auxiliary operators on LacI mediated repression of T7lacO promoters (in vivo). A) illustrates promoter sequences containing T7lacO promoters with auxiliary operator sequences of different strengths. B) Protein expression responses to 30 μM IPTG from the constructs depicted in A). GFP concentration units are expressed as µM/OD_600_. C) Dose responses to IPTG from the different constructs. Fluorescence response values are normalized to cell counts as determined by optical density values. Error bars depict standard deviation of triplicate measurements. Lines depict nonlinear regression fits to the Hill equation.

**Figure 3:**
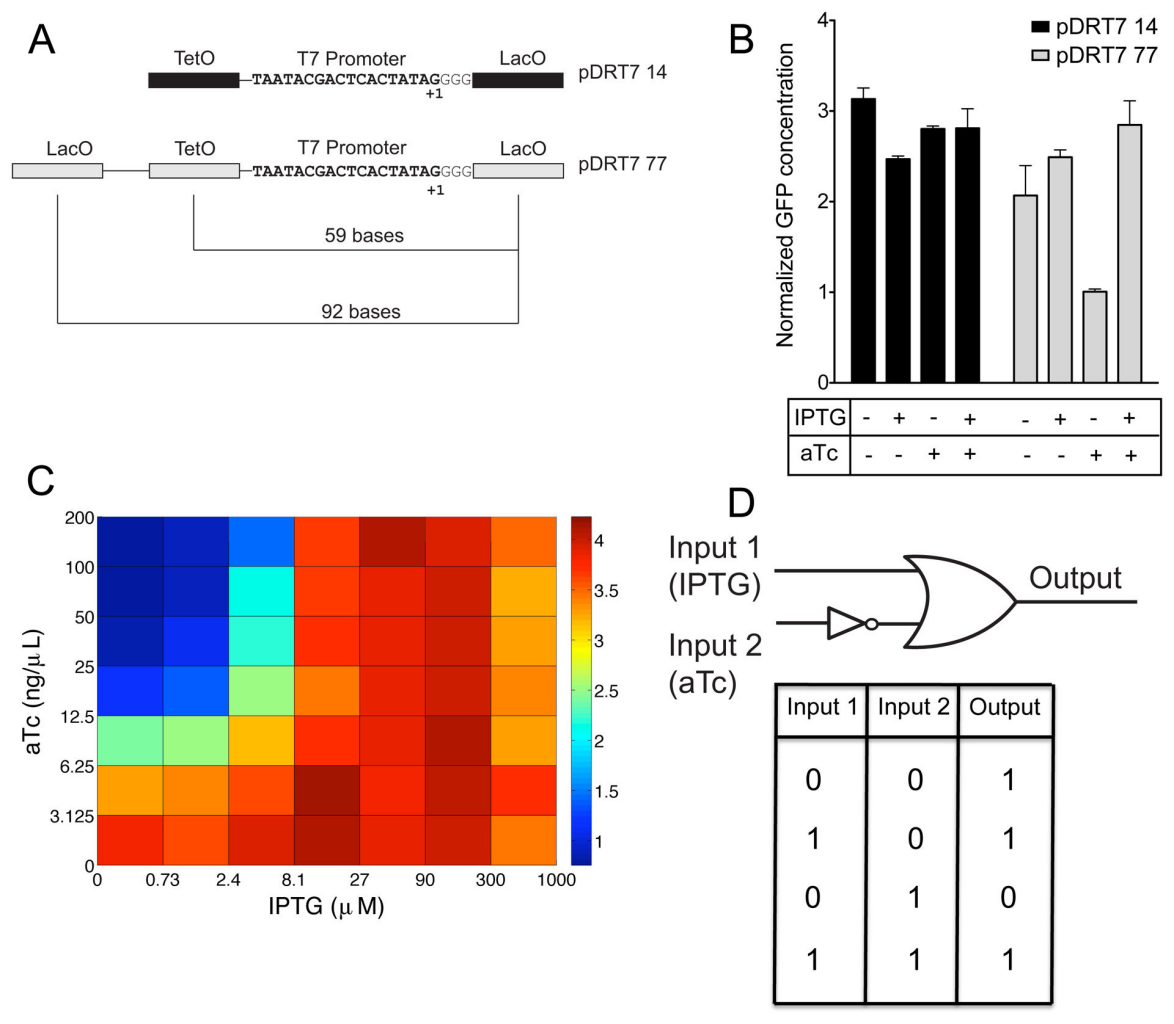
Effect of tetO on LacI mediated repression of T7lacO when tetO is in between the two lac operators (in vivo). Shown in (A) are the plasmid constructs pDRT7 14 and pDRT7 77. B) Displays the responses of these plasmids to presence /absence of 30 μM IPTG and 200 ng/ml aTc. C) Gene expression response, as determined by the normalized fluorescence response, of the pDRT7 77 plasmid to a range of IPTG and aTc concentrations. aTc concentration (ng/mL) is displayed on the X axis and the Y-axis denotes IPTG concentrations (μM). GFP fluorescence measurements in B and C are expressed as µM/OD_600_. D) is a schematic of the IMPLIES logic gate realized using the pDRT7 77 plasmid. Error bars depict standard deviation of triplicate measurements.

Enhanced GFP (eGFP)[32] expressing plasmids bearing different T7lacO and tet operator regions were co-transformed along with pTetRLacI plasmids into BL21-AI cells. A single colony from the transformation plate was used to initiate an overnight culture in LB media. A small aliquot of overnight culture was then transferred into M9 minimal media supplemented with 100 µg/mL ampicillin and 50 µg/mL kanamycin. The culture was incubated at 37°C for ~5 hours before this starter culture was again diluted in M9 media to a final optical density (OD_600_) of 0.01. 0.02% L-arabinose was added to the culture to induce the expression of T7 RNA polymerase. 100 µL aliquots of culture were dispensed into a 96-well plate (Corning 3370). Subsequently, IPTG and aTc were added to the wells as indicated. 50 μl of mineral oil was added to each of these wells to prevent drying of the samples. Absorbance (at 600 nm wavelength) and fluorescence measurements (485/20 nm excitation, 528/20 nm emission) were made at intervals of 7 minutes in a BioTek Synergy 2 plate reader. Fluorescence values indicated in Figures 2 and 3 represent measurements made at 200 minutes. Fluorescence values were corrected for background fluorescence of the media and cells in the absence of L-arabinose. Absorbance readings at 600 nm were used to normalize for cell density. Error bars in all the graphs represent standard deviation from three technical replicates.

### Cell-free expression experiments

**Figure 4:**
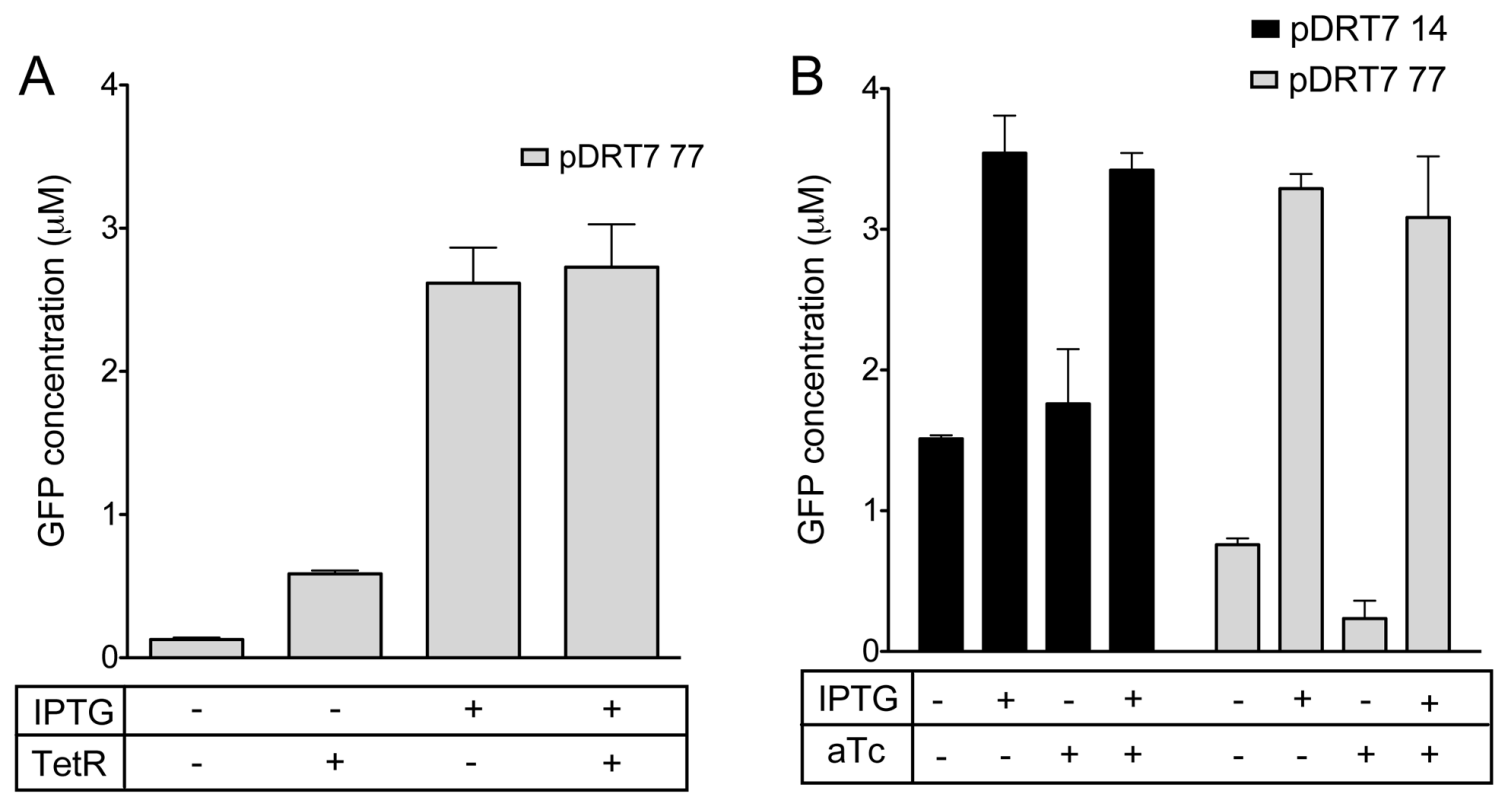
Effect of tetO on LacI mediated repression of T7lacO when tetO is in between the two lac operators in cell free systems. A) Fluorescence response from pDRT7 77 to LacI and TetR proteins. B) Shows fluorescence response from pDRT7 14 and pDRT7 77 plasmids to presence of 300 μM IPTG and/or 200ng/ml aTc. Error bars in the figure depict standard deviations of triplicate measurements.

Qiagen EasyXpress Protein Synthesis Kits were used for carrying out cell-free protein synthesis reactions. This kit was chosen because it does not contain IPTG and therefore would not interfere with the testing of the promoters designed in this study. In addition, the extract supplies LacI in concentrations sufficient for repression of the T7lacO promoter based constructs. Reactions were set up following the manufacturer’s instructions, but the final reaction volume was scaled down to 15 μL. The reactions were overlaid with 10 μL mineral oil to prevent drying. 300 μM IPTG and 200 ng/mL aTc were added to the reactions to induce expression. Reactions were set up in Corning CLS3820 plates, with the temperature set to 30°C. Fluorescence measurements were made at an interval of 7 minutes in a Biotek Synergy 2 plate reader. The fluorescence units shown in Figure 4 represent values obtained after 6 hours. In Figure 4 purified TetR was added to a final concentration of 1 μM. Concentrations of DNA and TetR used were based on previous optimizations [24]. The fluorescence values represent an average of three measurements and the error bars indicate the standard deviation.

## Results

### Effect of auxiliary operators upstream of the T7lacO promoter on LacI mediated repression

Most existing T7lacO promoters contain the lacO1 operator 4 bases from the transcriptional start site [23,25]. Since the presence of additional lac operators upstream of *E. coli* promoters are known to enhance LacI mediated repression from native *E. coli* lac promoters, we sought to examine if the presence of appropriately spaced lac operators would enhance repression from T7lacO promoters. (Figure 1A). Therefore, a LacO1 operator was first placed proximal to and downstream of the transcriptional start site of a T7 promoter that drives expression of enhanced Green Fluorescent Protein (eGFP) to construct pREPT7 01 (Figure 2A). To examine the effects of auxiliary lac operators on repression of T7lacO promoters, lac operators with different affinities for LacI were then placed 92 bases upstream of the primary LacO1 operator. This spacing places the operators in phase to form a DNA loop [33]. The interoperator distances described in this text indicates the number of bases separating the centre of the operators. LacO1, LacOID, with a greater affinity for LacI than LacO1, and LacO3, which is a weaker LacI binder than LacO1, were used as auxiliary operators[34] and placed 92 bases upstream of the T7lacO1 promoter to construct pREPT7 11, pREPT7 ID1 and pREPT7 31 respectively (Figure 2A). Each of these plasmids was co-transformed into BL21-AI *E. coli* cells (Invitrogen, CA) along with a pTetRLacI plasmid that expresses tet (TetR) and lac (LacI) repressors from a constitutive *E. coli* promoter. L-Arabinose was added to induce T7 polymerase expression, and the response of the promoters to LacI was measured by monitoring fluorescence changes in response to isopropyl β-D-1-thiogalactopyranoside (IPTG), which is a negative regulator of the Lac repressor.

While the T7lacO promoter alone can enable LacI mediated transcriptional repression, it is effective in reducing the basal level expression only upon substantial accumulation of LacI in the cell (Figure S1). In contrast, the presence of the auxiliary operator can increase repression ~4.5 fold when compared to the standard T7lacO1 operator (Figure 2B). In addition, reducing the L-arabinose concentrations from 0.2% (as recommended by the manufacturer), to 0.02% resulted in a greater fold change in gene expression upon L-arabinose addition (Figure S2). Moreover, induction of T7 RNA polymerase expression using 1/10^th^ the concentration required for maximal induction averted fitness effects typically encountered while using T7 promoter based systems for protein expression (Tables S2 and S3). As shown in Figure 2B, while LacI mediated repression levels from pREPT7 ID1 were found to be similar to pREPT7 11, repression levels from pREPT7 31 were similar to pREPT7 01, which does not have a distal lac operator site (Figure 2B and 2C). These results indicate that the presence of a strong distal operator is essential for enhancing LacI mediated repression of T7lacO1 promoters. Further, these results indicate that the strength of the upstream operator can be tuned to modulate repression levels from T7lacO promoters. Figure 2C shows the dose response curve for the four constructs tested at different IPTG concentrations. For the concentrations tested, the responses of pREPT7 ID1 and pREPT7 11 were found to exhibit a sigmoidal response with a Hill coefficient of ~1.85 and 2.4 with half maximal concentrations of 2.292 ±0.25 and 3.5±0.203 μM, respectively. Overall, these results show that the presence of an auxiliary operator with high affinity for LacI 92 bases upstream of the primary operator results in improved repression from T7lacO promoters. 

### Engineering dual regulation of T7promoters using LacI and TetR

To engineer T7lacO promoters that respond to both TetR and LacI, a tet operator (tetO) that TetR binds was inserted into the T7lacO framework. The tetO site was positioned such that it could interfere with the DNA looping process, thereby resulting in a multi-input responsive T7 promoter. We tested the effect of placing tetO at positions between the two lac operators, either at locations away from or overlapping the T7 promoter region (Figure 1B). To test the responses of these dual input promoters, the plasmids were co-transformed with pTetRLacI into BL21-AI cells, and the fluorescence response to the addition of anhydrotetracycline (aTc) and IPTG was measured. Previous studies with *E. coli* lac operators show that operators placed at spacing of 59, 70 and 92 were in phase to form a DNA loop [33]. We hypothesized that placing a tet operator at one of these spacing might prove effective in interfering with the LacI mediated DNA loop formed between the lac operators and therefore with lacI mediated repression. Accordingly, the tetO site was placed 59 bases upstream from the T7lacO promoter in pREPT7 11 to create pDRT7 77. For comparison, tetO was introduced 59 bases upstream to T7lacO operator in pREPT714, which does not have a distal lac operator (Figure 3A), to create pDRT7 14. The pDRT7 77 construct exhibited high fluorescence in the absence of both of the inducers or in the presence of IPTG, whereas addition of aTc reduced GFP expression. This response can be described by an IMPLIES gate where GFP response is high for the condition (IPTG OR (NOT aTc)). High GFP expression in the absence of both IPTG and aTc (Figure 3B) and low expression levels in the absence of IPTG and the presence of aTc suggests that TetR bound to tetO effectively interferes with LacI looping and relieves LacI dependent repression. In contrast to placing the tet operator upstream of the T7 promoter, placing the tet operator at locations overlapping the T7 promoter was not found to optimally elicit such a response. While placing the tet operators at positions that overlapped reduced the basal expression levels, binding of TetR at these positions did not appear to significantly interfere with LacI mediated repression (Figure S3).

 A more detailed characterization of the gate was carried out by testing the response of the construct to eight different IPTG concentrations ranging from 0 to a 1000 μM and eight different aTc concentrations ranging from 0 to 200 ng/ml. As shown in Figure 3C, the response is characterized by low GFP expression for IPTG concentrations below 2.4μM and over 6.25 ng/ml aTc. The gate showed a ~4.0 fold increase in expression for the rest of the aTc concentrations and for IPTG concentrations up to 300 μM. At higher concentrations of IPTG, the response was marked by a slight decrease in expression. Overall, the plasmid exhibited an IMPLIES gate (Figure 3D), and expression results indicate that TetR hinders LacI loop mediated repression. 

### Testing the dual input promoters in a cell-free expression system

The pDRT7 77 construct was tested further in cell-free extracts to quantify its response to LacI and TetR repressors. The commercial extract was chosen since it contains a cache of LacI protein. To enable TetR-mediated repression, 1μM of purified TetR protein was added to the reaction that contains 8 nM of DNA and the response of the multi-input promoters was measured by monitoring fluorescence changes upon the addition of IPTG and aTc. 

Figure 4A shows the response of pDRT7 77 to the addition of LacI alone and the addition of LacI in combination with purified TetR in a cell-free reaction. As expected, while expression is completely repressed in the presence of LacI, addition of purified TetR increased the expression by about 4.5 fold. Addition of concentrations of TetR higher than 1 μM did not increase expression from pDRT7 77 any further (data not shown). The responses of pDRT7 77 and pDRT7 14 (control without the auxiliary operator) to IPTG indicate that auxiliary operators increased LacI dependent repression by ~4 fold at the tested concentrations of LacI and template (Figure 4B). Furthermore, TetR was found to relieve LacI dependent repression, and expression levels were found to be similar to those from the constructs lacking the auxiliary operators. 

## Discussion

The field of synthetic biology has seen tremendous progress in the past few years and has led to the development of increasingly sophisticated gene networks. Several examples of transcriptional logic gates have been described using bacterial promoters, and transcriptional elements have been extensively used for construction of small circuits such as logic gates, oscillators and bistable switches. Although engineered control of gene expression is possible with bacterial promoters, implementation of orthogonal networks in bacterial systems is complicated by the shared use of the host’s machinery [35,36]. In contrast to bacterial promoters, however, the use of viral promoters offers a promising path to orthogonality if similar logic control systems can be established. Accordingly, T7 promoters have been used to construct a modular AND gate and to construct orthogonal feed forward loops in *E. coli* [14,22]. More recently, Shis and Bennet designed an AND gate using a split two component T7 RNA polymerase as an input [21]. 

However, the number of transcriptional logic control systems utilizing the T7 promoter is limited when compared to those described using bacterial promoters, which take advantage of the concerted action of multiple transcription factors. For instance, Isalan et.al described a T7 promoter regulated by two zinc finger proteins, which was constructed by placing two different operators that bind two zinc finger proteins[28]. Typical bacterial control elements bind cis-regulatory regions that can span more than 100 bp upstream and downstream to the transcription start site [37,38]. Consequently, a broad array of regulation mechanisms can be incorporated that operate either individually or in concert to create programmable, multiple input responsive promoters. As an illustration of the power of this approach, Hunziker and colleagues designed 12 different types of logic functions using a combination of cAMP-CRP activator protein, GalR repressor protein and promoters of diverse strengths [39]. However, the short cis-regulatory region around T7 promoters presents a challenge for implementing logic control using the aforementioned strategies. 

In contrast to cis-regulatory strategies, protein dimerization mediated DNA looping permits transcriptional control by proteins bound at locations distal to the promoter in both natural and synthetic promoters. In particular, the role of DNA looping for transcriptional regulation of bacterial promoters by *lac*[29,33], *araBAD*[40], *gal*[41] and *cI*[42] has been extensively studied in prokaryotes[43]. Additionally, in native *E. coli* lac promoter systems, looping facilitates tight repression of *E. coli* lac promoters even at low LacI repressor concentrations[44]. DNA looping has also been employed for constructing logic gates. Zhan et al. utilized two different lacO binding sites with *E. coli* promoters to introduce looping, thereby enabling regulation by the concerted action of two different repressors bound at different sites[45]. 

 Here, we implemented an IMPLIES gate using a strategy that harnesses DNA looping. While the IMPLIES gate is not commonly used in electrical engineering, the value of constructing an IMPLIES gate described here, lies in the fact that, IMPLIES gates together with NOT gates can be utilized to realize any logic function, as described in Figure S6. Therefore, the IMPLIES gate constructed here provides a powerful way forward for harnessing the orthogonality of T7 promoters to perform any desired logic operation. Here we addressed the challenging aspect of engineering dual control of T7 promoters. 

First, we assessed the potential advantages of DNA looping for control of the T7 promoter system. Without looping, very weak repression is observed, as shown by pREPT7 01 in Figure 2B. This may come as a surprise in light of the fact that strong overall induction of gene expression is achieved with the pET system [23]. However, in the pET system, IPTG simultaneously induces both T7 polymerase and the T7LacO promoter, which makes it difficult to assess the true performance of the T7lacO promoter. This is why a strain with arabinose inducible T7 polymerase was chosen. In addition, we induce cells at a low OD value (0.01) as compared to the typical OD values at which the pET system is induced. This can lead to weak accumulation of LacI prior to induction. After characterizing repression without looping, we then inserted LacO1 operator sequences around the T7 promoter. Specifically, a LacO1 auxiliary operator was placed 92 bases upstream from a primary LacO1 operator that resides immediately downstream of the T7 promoter. This architecture enabled strong repression of the T7 promoter in the presence of LacI. As shown in Figure 2B, introduction of an auxiliary LacI binding site resulted in ~3.5-fold increase in LacI mediated repression in comparison to T7lacO promoters lacking the auxiliary distal operator. Additionally, changing the auxiliary operator sequence such that LacI binding was weakened allowed tuning of repression levels from T7lacO promoters. These results mirror the effect of auxiliary operators on expression from *E. coli* lac promoters[29] and demonstrate the ability to further regulate expression from T7 promoters.

A previous investigation of DNA looping for LacI mediated control of the T7 promoter placed the auxiliary operator 238 bp away from T7lacO promoter, which resulted in only modest repression [23]. The enhanced repression levels reported here likely result from the use of a shorter distance between lac operators[44]. In studies based on *E. coli* lac promoters, a substantial decrease in repression levels accompanied an increase in operator distance [33]. A 50-fold change in gene expression was observed at an inter-operator distance of 70.5, while only a 15-fold change in expression was found when the distance was 150 bp. At shorter distances, the energy required for formation of a LacI dependent loop between operators located on the same side of the DNA helix is lower than the energy required for formation of a loop between operators that are out of phase[30,44]. Therefore, for the constructs pDRT7 77 and pDRT7 70 (described in Table 1 and Table S1) the inter-operator distances of 92 and 70 were chosen based on *in vivo* data for LacI mediated transcriptional control with *E. coli* promoters. The dual binding site design leads to a significant increase in repression levels when compared to a single LacI binding site. In our T7 system, as observed with *E. coli* promoters, controlling the distance between protein binding sites may enable further tuning of T7lacO promoters (Figure S5).

Having established the value of placing additional lac operator site upstream to T7lacO promoter to regulate transcription, we next sought to place a second regulatory site that might interfere with the looping process thereby interfering with lacI mediated repression. Accordingly, a tet operator was placed 59 bases upstream from the primary lac operator. This positioning of tetO resulted in a 2 to 3 fold change in gene expression upon the addition of aTc and in the absence of IPTG (Figure 3B-C). In contrast, inserting tetO within the LacO looping framework, at -21, -23 and -25 positions resulted in little to no observable reduction in expression in the presence of aTc and in the absence of IPTG when compared to the uninduced state (Figure S4). These results suggest that while TetR bound at tetO centered at the -59 position is optimally placed to interfere with LacI mediated repression of the T7lacO promoter by interfering with the formation of the LacI mediated DNA loop and therefore with LacI mediated repression.

The differences in the response of pDRT7 77, pDRT7 21, pDRT7 23, pDRT7 25 and pDRT7 27 (Table S1) to aTc (Figure 3B and Figure S3) may be explained by the orientation of tetO binding site relative to the DNA loop formed between the two lac operators. The ability of TetR to sterically hinder the formation of the LacI based loop may depend on position. In the case of *E. coli* promoters, a periodic dependence on distance between the lac operators has been observed [29,33]. A repression maxima is seen for distances that place the operators in phase and occurs with a periodicity of 11 to 11.3 bp with *E. coli* promoters of 59 bases, 70.5, 81.5 and 92 bases. Minima appear when the operators are placed on the opposite sides of DNA [30,33]. Based on this model, tetO centered 59 bp upstream to the primary lac operator would be in phase with the primary lac operator and may sterically hinder LacI mediated loop formation. Alternatively, TetR interference with LacI mediated repression could result from stiffening of the DNA template by TetR thereby increasing the persistence length of the DNA and making loop formation energetically unfavorable. Although confirmation of the underlying mechanism awaits further experiments, TetR binding to tetO at a distal site interferes with LacI dependent repression and enables an IMPLIES logic function. Moreover, we observe sharp transitions between on and off states. As shown in Figure 2, the Hill coefficient of response to IPTG for pREPT7 11 construct is ~2. Participation of protein tetramers for achieving repression likely contributes to the steepness of the response to IPTG. The result is IMPLIES logic behavior characterized by a sharp transition between the on and the off states.

We caution that, to fully capitalize on the orthogonality offered by T7 transcription, other system components must be chosen carefully with regards to the host genotype. Although we used well-characterized Lac system components that are derived from *E. coli*, host strains could be chosen to avoid potential interactions. Moreover, cell-free systems can be used to provide a minimal context with reduced chances for host interactions. 

The T7 promoter regulated by both LacI and TetR was further tested in cell extracts and constitutes a demonstration of digital logic in a cell-free system using T7 promoters. To test the logic gate, purified TetR was added to the extract, which already contained Lac repressor protein. As with the live cell systems, the presence of TetR protein interferes with LacI based repression, and an IMPLIES logic function is realized (Figure 4B).

Cell-free systems are ideal platforms for developing and implementing simple regulatory circuits. In contrast to the use of live cells, cell-free gene circuits offer the advantage of harnessing biological function without regard for cell survival. To optimize a gene circuit function, different components of the circuit such as the DNA sequence and the transcriptional regulator concentrations can be precisely defined to identify conditions for eliciting the desired response from a gene circuit. In contrast, in live cells these parameters can only be coarsely determined by modulating either the origin of replication to tune DNA copy number or other determinants of genetic expression for tuning regulator concentrations. Consequently, cell-free systems have tremendous potential for implementing predictable and precisely defined gene circuits, as they combine the simplicity of an *in vitro* system along with the remarkable capability for continuous protein production from DNA encoded instructions[24,46]. Further, control of biochemical components can be combined with defined physical platforms that mimic the size and scale of biological reactions[47]. Consequently, gene circuits and biosynthetic capabilities can be harnessed to evaluate gene circuits and biochemical reactions in cell-free environments [48-52]. Together, our demonstration of dual input regulation of T7 expression and digital logic contributes to a small but growing body of work for construction of more sophisticated cell-free circuits [18,24,53-56].

In future work, several approaches can be taken to optimizing the dynamic range of our components. A more detailed examination of lac operator spacing, as has been done with *E. coli* promoters, may facilitate tuning of the response to suit downstream application requirements. As described by Khalil et.al, transcriptional output can be tuned through systematic modification of operator count and operator affinity for the transcriptional factor (as shown in Figure 2) [57]. In addition, repressor concentrations may be tuned to optimize dynamic range. However, excessively large induction factors are not always required to construct interested gene circuits. For example, Tabor et al constructed a ‘dark sensor’ optogenetic component with a dynamic range of less than 3-fold yet proceeded to successfully integrate this with other components to construct a bacterial ‘edge detection’ circuit [58]. If necessary, dynamic range can also be expanded through the use of signal amplifying gene circuits, as explained and experimentally demonstrated [59]. Such amplification was realized in the construction of multicellular logic functions by Tamsir et al. [60]. 

The work described here marks a starting point for using T7 promoters to implement digital logic and other complex functionality in biochemical systems. As described in Figure S6, the IMPLIES gate constructed here may be combined with NOT gates to realize any logic function using T7 transcription. DNA looping harnessed in this strategy not only improves the repression of expression from T7 promoters but expands the otherwise limited region that can be used to exert control over T7 promoters. Overall, the approach put forth here, combined with the existing availability of promoters of varied strength[61], polymerase specificity[20,62] and two component T7 RNA polymerases[21] paves the way towards harnessing the portability of T7 promoters for realizing synthetic networks in cells and in cell-free systems that are more decoupled from cellular processes. 

## Supporting Information

Table S1 **Plasmids.** The plasmids used for experiments described in the supplementary information are described.
(DOCX)

Figure S1 **Effect of additional source of LacI.** Gene expression results from pREGT7 15b and pREPT7 11 encoded on pET3a backbones in response to addition of IPTG were carried out in order to examine the effect of the presence of an additional source of LacI in the cell. The use of pET15b backbone results in a higher concentration of LacI protein inside the cell as opposed to pREPT7 01 (pET3A backbone) which does not (manuscript Figure 2). The resulting plasmid pREGT7-15b was co-transformed with pTetRLacI into BL21-AI cells. The response of this plasmid to 30 µM of IPTG was compared to the response from pREGT7 77. Repression levels from T7lacO promoters encoded in the pET15b backbone were found to be similar to repression levels from T7lacO promoters with auxiliary lacO operators encoded on a backbone lacking the additional source of *lacI*. The GFP concentration units are expressed as µM/OD_600_.
(TIFF)

Figure S2 **Effect of arabinose concentration on repression from pREPT7 11.** The effect of adding different concentrations of arabinose to induce T7 RNA polymerase concentrations on repression levels from T7lacO promoters with auxiliary operators was examined. pREPT7 11 was co- transformed with pTetRLacI into BL21-AI cells and T7 RNA polymerase expression was induced by 0.02%, 0.1% and 0.2% L-arabinose. The protein expression response to 30 µM IPTG was determined using a plate reader. An increase in repression levels results from reduction in arabinose concentrations. A 1.4 fold induction is observed in the presence of IPTG when the arabinose concentration is 0.2%. A 5-fold induction is observed when the IPTG concentration is reduced to 0.02%. The data shown are fluorescence responses from pREPT7 11 plasmids to three different arabinose concentrations after 200 minutes. GFP concentration units are expressed µM/OD_600_.
(TIFF)

Figure S3 **Effect of placing tet operator at positions overlapping the promoter.** The effect of placing TetR binding sites at positions overlapping the T7 promoter was examined. The upper panel (A) illustrates the constructs pDRT7 21, pDRT7 23, pDRT7 25 and pDRT7 27. The underlined text refers to the tetO operator sequence whereas T7 promoter sequence is depicted by bold text. The graphs in the lower panel (B) indicate the expression response of these plasmids in the presence/absence of 30 µM IPTG and 200ng/ml aTc. The normalized GFP concentration units are expressed units of µM/OD_600_.
(TIFF)

Information S1 **Effect of placing tet operator at positions overlapping the promoter.**
(DOCX)

Figure S4 **Time course of expression from truncated promoters.** Protein expression time courses for the constructs pDRT7 21, pDRT7 23, pDRT7 25 and pDRT7 27 in the absence and presence of 30 μM IPTG are described. These truncated promoters had different effects on LacI mediated repression when considered in isolation from TetR repression system. In the absence of IPTG, while the expression from pDRT7 21, pDRT7 23 and pDRT7 25 remains low at time points after 200 minutes, pDRT7 27 appears strong at later time points due to leaky repression from T7lacO. Moreover, the highly processive T7 RNA polymerase can generate large enough amounts of RNA transcripts to saturate the translational machinery[63] and therefore mask the repression that occurs initially. In contrast, the truncated versions of the T7 promoters are less efficient and generate lower amounts of transcript. This avoids saturating the expression system, resulting in noticeable repression levels even at later times. Consequently, tight control and a wide range of repression levels can be achieved when truncated versions of T7 promoters are combined with regulatory sequences. This behavior likely results from saturation of the translation machinery by high levels of transcript generated from the intact promoter. The graph depicts results from 16 hours of expression. The graphs indicate fluorescence values corrected for background and normalized to optical density readings.
(TIFF)

Figure S5 **Effect of distance between lac operators on LacI mediated repression.** The effect of changing lacO inter-operator distance on LacI and Tet mediated repression was examined. The distance between the lac operators was shortened to 70 bases while retaining the tetO at the -27 position to yield pDRT7 70. Under the conditions tested, LacI mediated repression from this construct was not significantly different from pDRT7 27. However, as with pDRT7 27, TetR negatively regulated LacI mediated repression. Both the distance and the relative phasing between the lac operators have an effect on the efficiency of LacI mediated repression and that an inter-operator distance of 70 bp achieves the strongest repression of *E. coli* Lac promoters[29,30]. The results shown in Figure S5 indicate that reducing the distance between the lac operators from 92 bp in pDRT7 27 to 70bp in pDRT7 70 did not significantly change LacI mediated repression levels from T7lacO promoters. However, TetR continues to interfere with LacI mediated repression as indicated by lower expression levels in the presence of aTc and the absence of IPTG when compared to the uninduced state. A) depicts the pDRT7 27 and pDRT7 70 constructs used in this experiment. The underlined text indicates the tet operator sequence while the bold text corresponds to the T7 promoter sequence. B) Responses of pDRT7 27 and pDRT7 70 to 30 μM IPTG and 200ng/ml aTc concentrations. The normalized GFP concentration units are expressed in units of µM/OD_600_.
(TIFF)

Figure S6 **Potential logic gates that can be constructed from IMPLIES gate.** The design of NOT, AND, and OR gates from our experimentally characterized gates are shown. The described dual input T7 regulation approach lends itself to the simple construction of IMPLIES gates. IMPLIES gates in conjunction with NOT gates can together be used to implement any logic function. Shown are how AND and OR gates can be constructed from IMPLIES and NOT gates. Letting I(a,b) represent the IMPLIES function of a and b, where I(a,b)=a’+b, an AND gate can be constructed as ab=I(a,b’)’ and an OR gate can be constructed as I(a’,b). Genetic implementations are shown in the figure, where a, b, c, and d are different repressor proteins, and o(a), o(b), o(c), and o(d) represent the respective operator sites for each of these repressor proteins.
(TIFF)

Table S2 **Absorbance values for experiments described in Figure 2C.** Fitness effects must be carefully considered in live cell experiments, particularly with the use of the highly processive T7 RNA polymerase, which can potentially overwhelm the host’s expression machinery. In the described live *E. coli* experiments, inducing T7 RNA polymerase at low levels averts this toxicity issue. Specifically, T7 RNA polymerase was induced with 0.02% arabinose, which is approximately 1/10^th^ the concentration required for maximal induction. Tables S2 and S3 depict absorbance readings at a wavelength of 600 nm and show that culture density does not vary strongly under the different inducer conditions.
(DOCX)

Table S3 **Absorbance values for experiments described in Figure 3B.**
(DOCX)

Information S2 **Sequences of regulatory region.** In the described sequences, the blue colored bases indicate the Lac operator, the brown indicates the tet operator, the black underlined text corresponds to the T7 promoter, the red sequences are the ribosome binding site and the red underlined text corresponds to the T7 terminator.
(DOCX)
