# Supplementary material for: Multi-Input Regulation and Logic with T7 Promoters in Cells and Cell-Free Systems
Source: PLoS One. 2013 Oct 23;8(10):e78442. doi: 10.1371/journal.pone.0078442 (PMC3806817; doi:10.1371/journal.pone.0078442)
Supplement: Table S1 — Plasmids. The plasmids used for experiments described in the supplementary information are described. (DOCX) [file pone.0078442.s001.docx]

**Table S1. Plasmids**

| Plasmid Name | Promoter | Operator, position | Gene | Plasmid Backbone* |
| --- | --- | --- | --- | --- |
| pREGT7-15b | T7lacO | LacO1, +14 | *EGFP* | pET15b |
| pDRT7 70 | T7lacO | LacO1, +14,- 55  tetO ,-27 | *EGFP* | pET3a |
| pDRT7 21 | T7_-6_lacO | LacO1, +14,- 77  tetO, -21 | *EGFP* | pET3a |
| pDRT7 23 | T7_-4_lacO | LacO1, +14, -77  tetO, -23 | *EGFP* | pET3a |
| pDRT7 25 | T7_-2_lacO | LacO1, +14, -77  tetO, -25 | *EGFP* | pET3a |
| pDRT7 27 | T7lacO | LacO1, +14, -77  tetO, -27 | *EGFP* | pET3a |

* pET15b and pET3a have copy numbers of approximately 40 [[1](#_ENREF_1)], and pPROLAR has a copy number of 20-30 [[2](#_ENREF_2)].

1. Milo R, Jorgensen P, Moran U, Weber G, Springer M (2010) BioNumbers—the database of key numbers in molecular and cell biology. Nucleic acids research 38: D750-D753.

2. Lutz R, Bujard H (1997) Independent and tight regulation of transcriptional units in Escherichia coli via the LacR/O, the TetR/O and AraC/I1-I2 regulatory elements. Nucleic acids research 25: 1203-1210.
