# Supplementary figures and images for: Multi-Input Regulation and Logic with T7 Promoters in Cells and Cell-Free Systems
Source: PLoS One. 2013 Oct 23;8(10):e78442. doi: 10.1371/journal.pone.0078442 (PMC3806817; doi:10.1371/journal.pone.0078442)

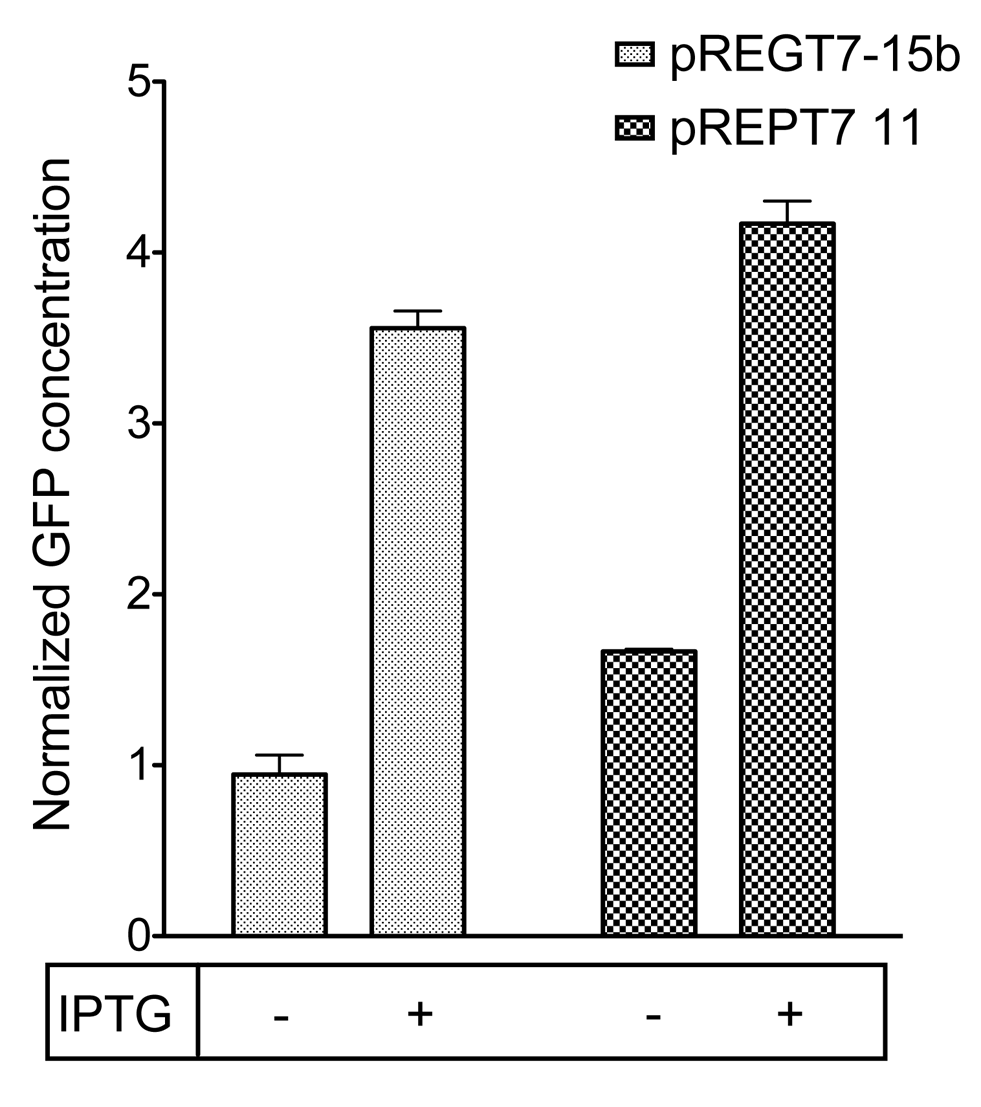

Supplement: Figure S1 — Effect of additional source of LacI. Gene expression results from pREGT7 15b and pREPT7 11 encoded on pET3a backbones in response to addition of IPTG were carried out in order to examine the effect of the presence of an additional source of LacI in the cell. The use of pET15b backbone results in a higher concentration of LacI protein inside the cell as opposed to pREPT7 01 (pET3A backbone) which does not (manuscript Figure 2). The resulting plasmid pREGT7-15b was co-transformed with pTetRLacI into BL21-AI cells. The response of this plasmid to 30 µM of IPTG was compared to the response from pREGT7 77. Repression levels from T7lacO promoters encoded in the pET15b backbone were found to be similar to repression levels from T7lacO promoters with auxiliary lacO operators encoded on a backbone lacking the additional source of lacI. The GFP concentration units are expressed as µM/OD600. (TIFF) [file pone.0078442.s002.tiff]

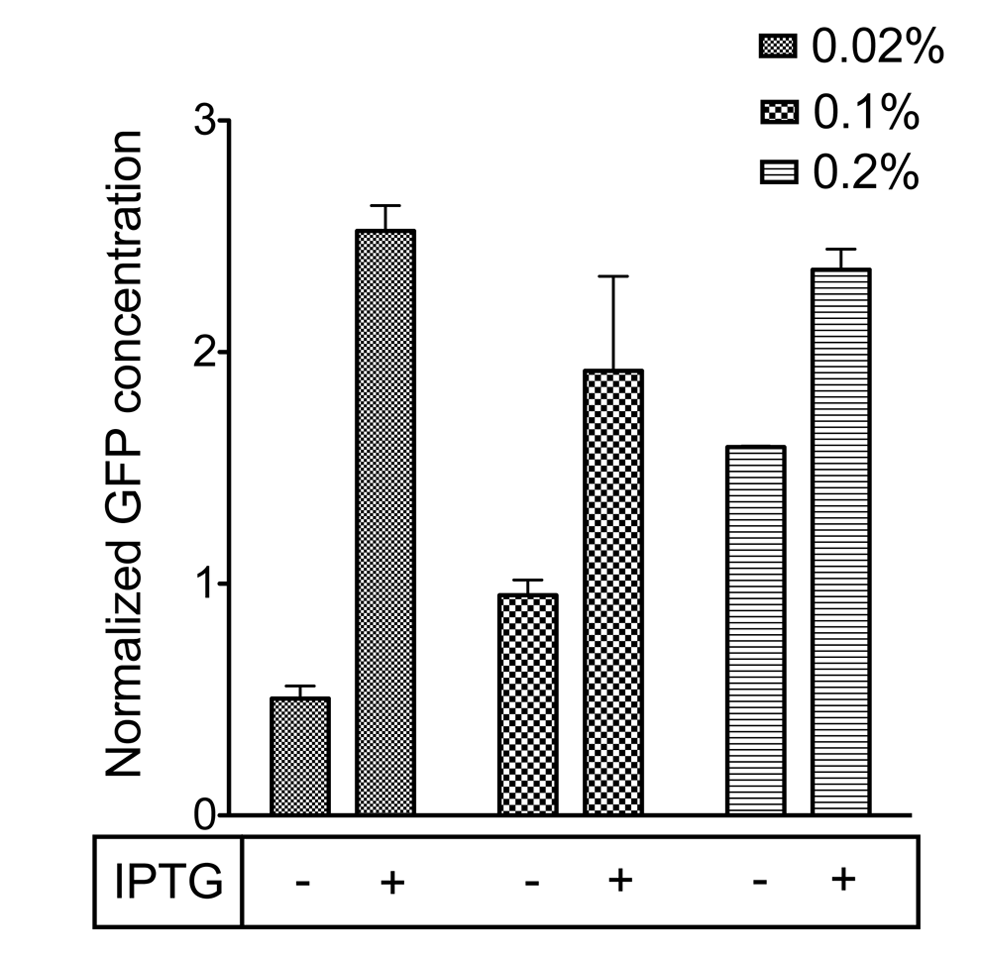

Supplement: Figure S2 — Effect of arabinose concentration on repression from pREPT7 11. The effect of adding different concentrations of arabinose to induce T7 RNA polymerase concentrations on repression levels from T7lacO promoters with auxiliary operators was examined. pREPT7 11 was co- transformed with pTetRLacI into BL21-AI cells and T7 RNA polymerase expression was induced by 0.02%, 0.1% and 0.2% L-arabinose. The protein expression response to 30 µM IPTG was determined using a plate reader. An increase in repression levels results from reduction in arabinose concentrations. A 1.4 fold induction is observed in the presence of IPTG when the arabinose concentration is 0.2%. A 5-fold induction is observed when the IPTG concentration is reduced to 0.02%. The data shown are fluorescence responses from pREPT7 11 plasmids to three different arabinose concentrations after 200 minutes. GFP concentration units are expressed µM/OD600. (TIFF) [file pone.0078442.s003.tiff]

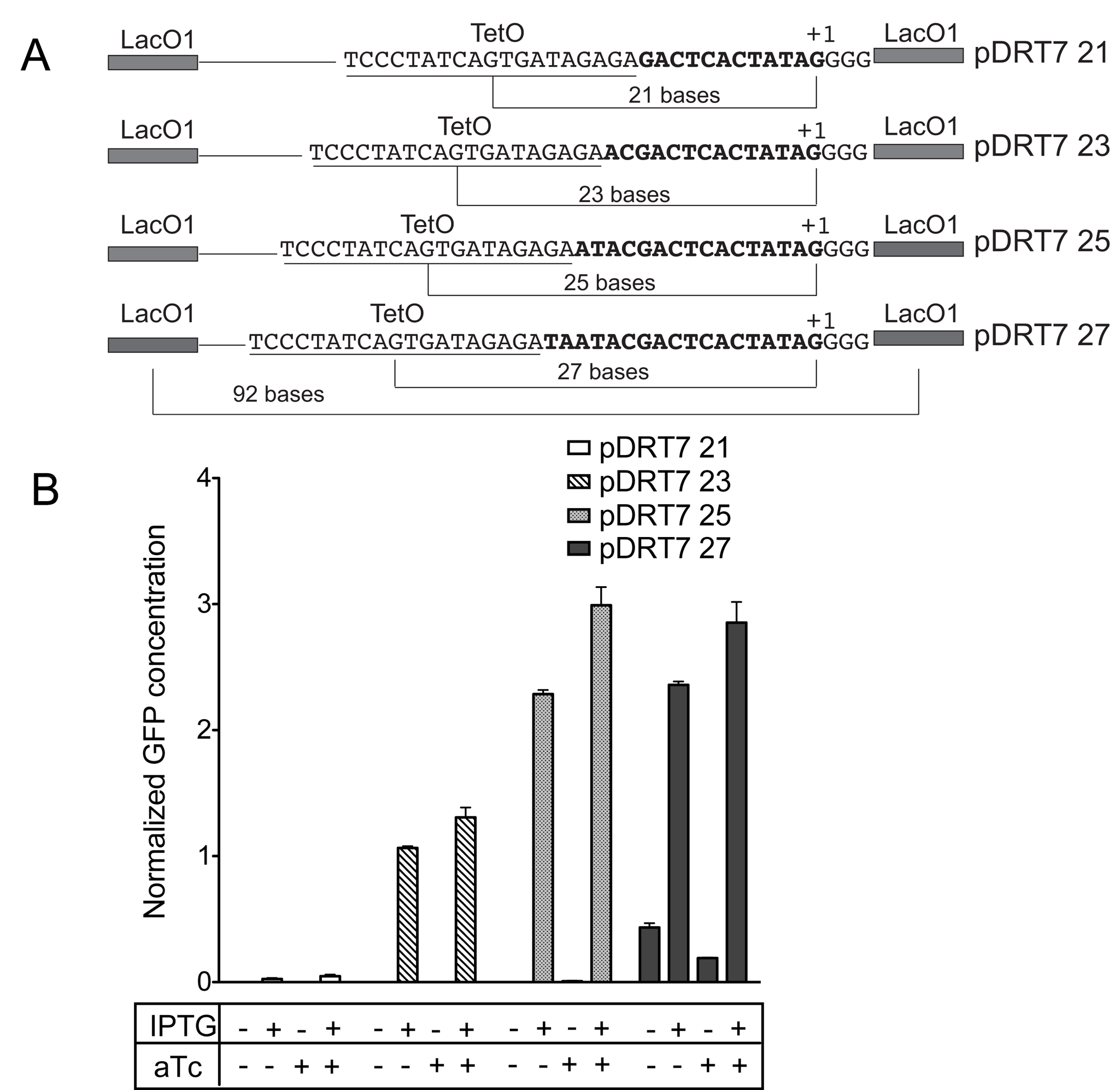

Supplement: Figure S3 — Effect of placing tet operator at positions overlapping the promoter. The effect of placing TetR binding sites at positions overlapping the T7 promoter was examined. The upper panel (A) illustrates the constructs pDRT7 21, pDRT7 23, pDRT7 25 and pDRT7 27. The underlined text refers to the tetO operator sequence whereas T7 promoter sequence is depicted by bold text. The graphs in the lower panel (B) indicate the expression response of these plasmids in the presence/absence of 30 µM IPTG and 200ng/ml aTc. The normalized GFP concentration units are expressed units of µM/OD600. (TIFF) [file pone.0078442.s004.tiff]

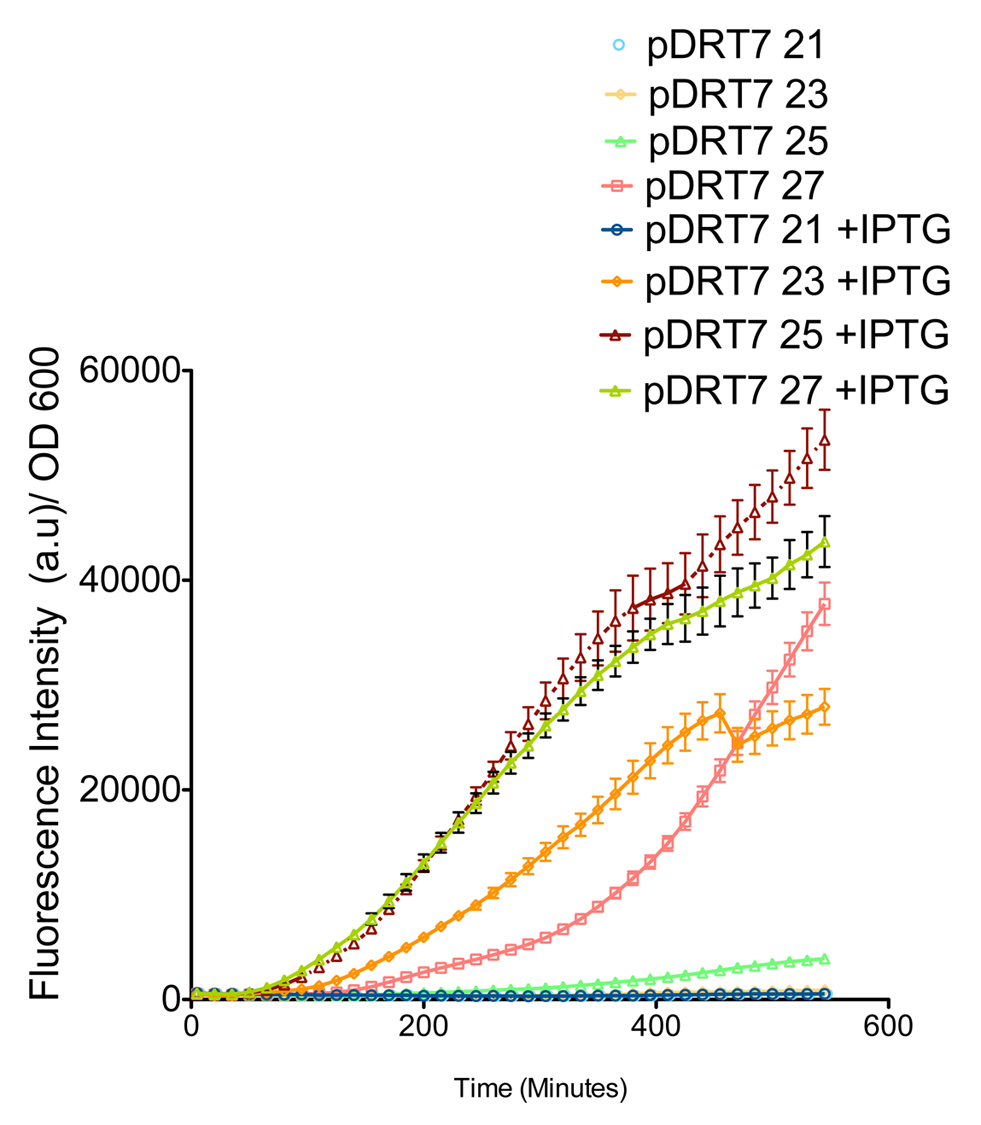

Supplement: Figure S4 — Time course of expression from truncated promoters. Protein expression time courses for the constructs pDRT7 21, pDRT7 23, pDRT7 25 and pDRT7 27 in the absence and presence of 30 μM IPTG are described. These truncated promoters had different effects on LacI mediated repression when considered in isolation from TetR repression system. In the absence of IPTG, while the expression from pDRT7 21, pDRT7 23 and pDRT7 25 remains low at time points after 200 minutes, pDRT7 27 appears strong at later time points due to leaky repression from T7lacO. Moreover, the highly processive T7 RNA polymerase can generate large enough amounts of RNA transcripts to saturate the translational machinery[63] and therefore mask the repression that occurs initially. In contrast, the truncated versions of the T7 promoters are less efficient and generate lower amounts of transcript. This avoids saturating the expression system, resulting in noticeable repression levels even at later times. Consequently, tight control and a wide range of repression levels can be achieved when truncated versions of T7 promoters are combined with regulatory sequences. This behavior likely results from saturation of the translation machinery by high levels of transcript generated from the intact promoter. The graph depicts results from 16 hours of expression. The graphs indicate fluorescence values corrected for background and normalized to optical density readings. (TIFF) [file pone.0078442.s006.tiff]

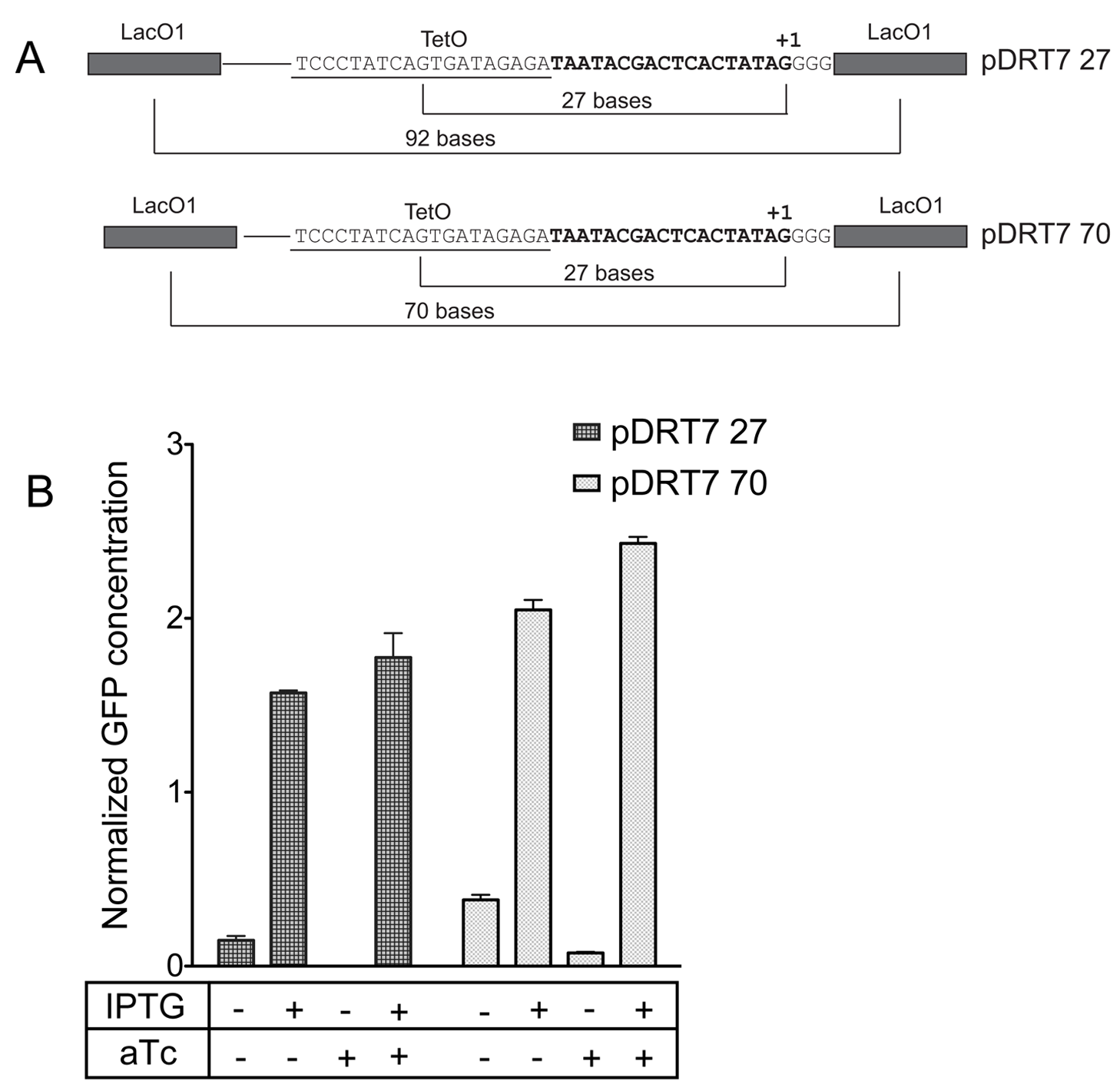

Supplement: Figure S5 — Effect of distance between lac operators on LacI mediated repression. The effect of changing lacO inter-operator distance on LacI and Tet mediated repression was examined. The distance between the lac operators was shortened to 70 bases while retaining the tetO at the -27 position to yield pDRT7 70. Under the conditions tested, LacI mediated repression from this construct was not significantly different from pDRT7 27. However, as with pDRT7 27, TetR negatively regulated LacI mediated repression. Both the distance and the relative phasing between the lac operators have an effect on the efficiency of LacI mediated repression and that an inter-operator distance of 70 bp achieves the strongest repression of E. coli Lac promoters[29,30]. The results shown in Figure S5 indicate that reducing the distance between the lac operators from 92 bp in pDRT7 27 to 70bp in pDRT7 70 did not significantly change LacI mediated repression levels from T7lacO promoters. However, TetR continues to interfere with LacI mediated repression as indicated by lower expression levels in the presence of aTc and the absence of IPTG when compared to the uninduced state. A) depicts the pDRT7 27 and pDRT7 70 constructs used in this experiment. The underlined text indicates the tet operator sequence while the bold text corresponds to the T7 promoter sequence. B) Responses of pDRT7 27 and pDRT7 70 to 30 μM IPTG and 200ng/ml aTc concentrations. The normalized GFP concentration units are expressed in units of µM/OD600. (TIFF) [file pone.0078442.s007.tiff]

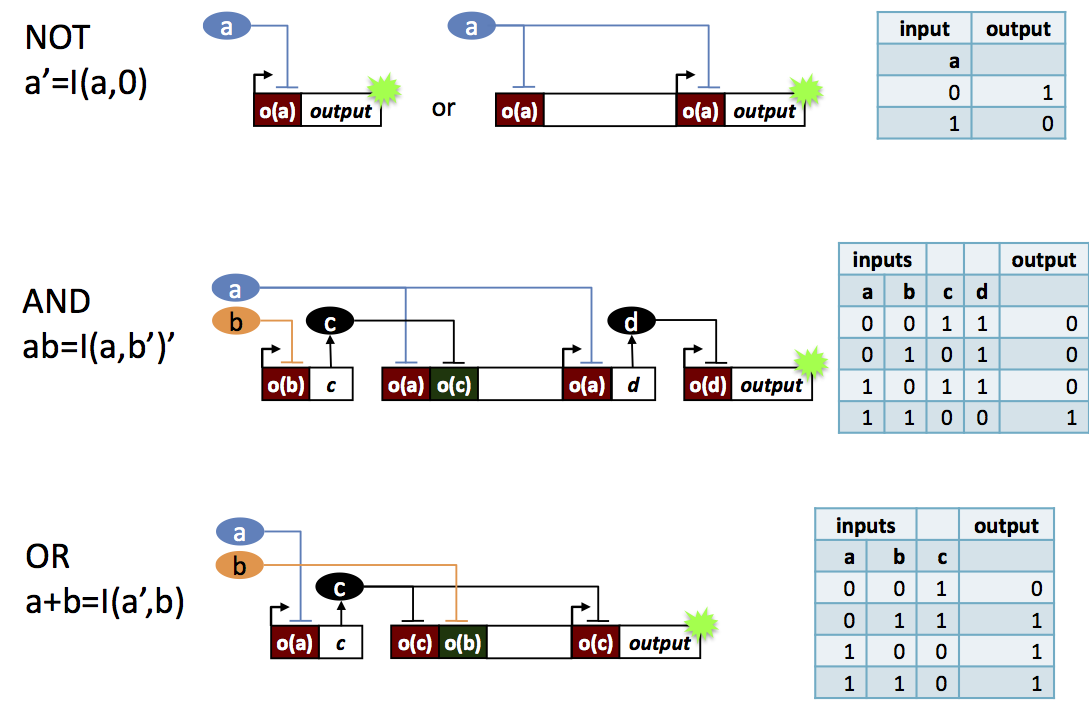

Supplement: Figure S6 — Potential logic gates that can be constructed from IMPLIES gate. The design of NOT, AND, and OR gates from our experimentally characterized gates are shown. The described dual input T7 regulation approach lends itself to the simple construction of IMPLIES gates. IMPLIES gates in conjunction with NOT gates can together be used to implement any logic function. Shown are how AND and OR gates can be constructed from IMPLIES and NOT gates. Letting I(a,b) represent the IMPLIES function of a and b, where I(a,b)=a’+b, an AND gate can be constructed as ab=I(a,b’)’ and an OR gate can be constructed as I(a’,b). Genetic implementations are shown in the figure, where a, b, c, and d are different repressor proteins, and o(a), o(b), o(c), and o(d) represent the respective operator sites for each of these repressor proteins. (TIFF) [file pone.0078442.s008.tiff]
