# Supplementary material for: Multi-Input Regulation and Logic with T7 Promoters in Cells and Cell-Free Systems
Source: PLoS One. 2013 Oct 23;8(10):e78442. doi: 10.1371/journal.pone.0078442 (PMC3806817; doi:10.1371/journal.pone.0078442)
Supplement: Information S1 — Effect of placing tet operator at positions overlapping the promoter. (DOCX) [file pone.0078442.s005.docx]

**Information S1: Effect of placing tet operator at positions overlapping the promoter**

Several studies show that, barring a few key positions on the T7 promoter, the binding site of the promoter can be mutated without loss of function at the expense of reduced transcriptional output [1,2]. Therefore, we tested the effect of replacing a portion of the T7 promoter with tetO on transcription efficiency. Four different plasmids were constructed in which the TetR binding site tetO was centered at 21, 23, 25 and 27 bases upstream from the transcriptional start site of the T7lacO promoter to create pDRT7 21, pDRT7 23, pDRT7 25 and pDRT7 27 respectively as depicted in Figure S3A. The construct pDRT7 27 contains tetO 27 bases upstream from the transcriptional start site; the full T7 promoter remains intact. On the other hand, pDRT7 25, pDRT7 23 and pDRT7 21 contain truncated versions of the T7 promoter with 15, 13 and 11 bases of the wild type T7 promoter remaining, respectively. As expected, truncated T7 promoters that result from replacing a section of the T7 promoter with the tet operator reduced the promoter strength and led to lower GFP expression. This is especially true for the T7 promoter in pDRT7 21, where the tetO sequence nearly abolishes promoter activity. This finding is consistent with earlier reports showing that a mutation at the -10 position inactivates the T7 promoter [3,4]. LacI mediated repression was observed for all of the constructs as evidenced by low GFP expression levels in the absence of IPTG (Figure S3B). Addition of aTc alone did not lead to a change in fluorescence levels when compared to expression levels in the absence of inducer, indicating that TetR did not repress expression when bound at the -21, -23, -25 and -27 positions. However, when TetR is bound at the -27 position, which abuts the T7 promoter, some interference with LacI repression from the T7lacO repressors was observed and LacI based repression was incomplete in the presence of TetR. We note that the tet operator at -27 position is located 40 bases upstream from the primary lacO. This indicates that while TetR bound at tetO positioned at the -27 position interferes with LacI mediated repression of T7lacO promoters, it might not be optimally placed to relieve the repression completely.

1. Chapman KA, Burgess RR (1987) Construction of bacteriophage T7 late promoters with point mutations and characterization by in vitro transcription properties. Nucleic Acids Res 15: 5413-5432.
2. Martin CT, Coleman JE (1987) Kinetic analysis of T7 RNA polymerase-promoter interactions with small synthetic promoters. Biochemistry 26: 2690-2696.
3. Imburgio D, Rong M, Ma K, McAllister WT (2000) Studies of promoter recognition and start site selection by T7 RNA polymerase using a comprehensive collection of promoter variants. Biochemistry 39: 10419-10430.
4. Ikeda RA (1992) The efficiency of promoter clearance distinguishes T7 class II and class III promoters. J Biol Chem 267: 11322-11328.
