# Supplementary material for: Multi-Input Regulation and Logic with T7 Promoters in Cells and Cell-Free Systems
Source: PLoS One. 2013 Oct 23;8(10):e78442. doi: 10.1371/journal.pone.0078442 (PMC3806817; doi:10.1371/journal.pone.0078442)
Supplement: Table S2 — Absorbance values for experiments described in Figure 2C. Fitness effects must be carefully considered in live cell experiments, particularly with the use of the highly processive T7 RNA polymerase, which can potentially overwhelm the host’s expression machinery. In the described live E. coli experiments, inducing T7 RNA polymerase at low levels averts this toxicity issue. Specifically, T7 RNA polymerase was induced with 0.02% arabinose, which is approximately 1/10th the concentration required for maximal induction. Tables S2 and S3 depict absorbance readings at a wavelength of 600 nm and show that culture density does not vary strongly under the different inducer conditions. (DOCX) [file pone.0078442.s009.docx]

**Table S2: Absorbance values for experiments described in Figure 2C**

| IPTG (μM) | 0 | 1 | 3 | 10 | 30 |
| --- | --- | --- | --- | --- | --- |
| pREPT7 01 | 0.10 | 0.09 | 0.09 | 0.08 | 0.09 |
| pREPT7 11 | 0.11 | 0.11 | 0.12 | 0.09 | 0.08 |
| pREPT7 31 | 0.12 | 0.12 | 0.10 | 0.09 | 0.09 |
| pREPT7 ID1 | 0.12 | 0.13 | 0.12 | 0.10 | 0.10 |
