# Supplementary material for: Multi-Input Regulation and Logic with T7 Promoters in Cells and Cell-Free Systems
Source: PLoS One. 2013 Oct 23;8(10):e78442. doi: 10.1371/journal.pone.0078442 (PMC3806817; doi:10.1371/journal.pone.0078442)
Supplement: Table S3 — Absorbance values for experiments described in Figure 3B. (DOCX) [file pone.0078442.s010.docx]

**Table S3: Absorbance values for experiments described in Figure 3B**

|  | -/- | +/- | -/+ | +/+ |
| --- | --- | --- | --- | --- |
| pDRT7 14 | 0.09 | 0.10 | 0.08 | 0.09 |
| pDRT7 77 | 0.08 | 0.08 | 0.07 | 0.07 |
